# A chemical-genetics approach to study the role of atypical Protein Kinase C in *Drosophila*

**DOI:** 10.1242/dev.170589

**Published:** 2019-01-29

**Authors:** Matthew Hannaford, Nicolas Loyer, Francesca Tonelli, Martin Zoltner, Jens Januschke

**Affiliations:** 1 Cell and Developmental Biology, School of Life Sciences, University of Dundee, Dow Street, Dundee DD5 1EH, UK; 2 MRC Protein Phosphorylation and Ubiquitylation Unit, School of Life Sciences, University of Dundee, Dow Street, Dundee DD5 1EH, UK; 3 Biological Chemistry and Drug Discovery, School of Life Sciences, University of Dundee, Dow Street, Dundee DD5 1EH, UK

**Keywords:** *Drosophila*, Asymmetric cell division, atypical Protein Kinase C, Chemical genetics, Neuroblasts

## Abstract

Studying the function of proteins using genetics in cycling cells is complicated by the fact that there is often a delay between gene inactivation and the time point of phenotypic analysis. This is particularly true when studying kinases that have pleiotropic functions and multiple substrates. *Drosophila* neuroblasts (NBs) are rapidly dividing stem cells and an important model system for the study of cell polarity. Mutations in multiple kinases cause NB polarity defects, but their precise functions at particular time points in the cell cycle are unknown. Here, we use chemical genetics and report the generation of an analogue-sensitive allele of *Drosophila* atypical Protein Kinase C (aPKC). We demonstrate that the resulting mutant aPKC kinase can be specifically inhibited *in vitro* and *in vivo*. Acute inhibition of aPKC during NB polarity establishment abolishes asymmetric localization of Miranda, whereas its inhibition during NB polarity maintenance does not in the time frame of normal mitosis. However, aPKC helps to sharpen the pattern of Miranda, by keeping it off the apical and lateral cortex after nuclear envelope breakdown.

## Abstract (precis)

**Summary:** The generation of an analogue-sensitive allele of aPKC in *Drosophila* allows dissection of the function of aPKC kinase activity with high specificity and temporal control.

## INTRODUCTION

The polarization of the cell cortex is a key mechanism for proper epithelial cell organization as well as for the production of different cell types through asymmetric cell division. The establishment of cell polarity often involves the evolutionarily conserved PAR-complex, which polarizes many cell types across species (Goldstein and Macara, 2007). This complex includes PAR-3 [Bazooka (Baz) in flies], PAR-6 and the serine/threonine kinase atypical Protein Kinase C (aPKC), and it specifies the polarity axis of a cell in many contexts. An important effector of this complex is aPKC, which can regulate substrates through phosphorylation. In this way cells can establish subcellular patterns of molecules along their axis of polarity (Goehring, 2014; Hoege and Hyman, 2013; Suzuki, 2006).

In the neural stem cells of the fly, called neuroblasts (NBs), the function of aPKC is important for asymmetric divisions that control cell fates (Atwood and Prehoda, 2009; Rolls et al., 2003; Wodarz et al., 2000). A key feature of NBs is the polarized localization of fate determinants to one cell pole during mitosis. Upon division, the two daughter cells each receive a different set of molecular information resulting in two different cell fates. Although several kinases, including aPKC, have been shown to play important roles in this process (Lee et al., 2006; Rolls et al., 2003; Tio et al., 2001; Wang et al., 2007, 2006; Wodarz et al., 2000), their precise function remain obscure. This is in part because larval NBs have a cell cycle time of ∼1 h (Homem et al., 2013). Therefore, particular functions at specific time points in the cell cycle cannot be easily dissected using genetic perturbation of kinases such as aPKC.

Existing models that aim to explain asymmetric fate determinant localization in NBs assume spatial differences of aPKC activity in mitotic NBs along the apico basal polarity axis (Atwood and Prehoda, 2009; Barros et al., 2003). aPKC is recruited to the apical pole in mitosis by Baz and subsequently activated. Once activated, aPKC phosphorylates different substrates including the fate determinants Miranda (Mira) and Numb. The phosphorylation occurs on a plasma membrane (PM) binding region such that phosphorylation disrupts membrane interaction (Bailey and Prehoda, 2015; Betschinger et al., 2005; Ikeshima-Kataoka et al., 1997; Knoblich et al., 1995; Peng et al., 2000; Petronczki and Knoblich, 2001; Rolls et al., 2003; Shen et al., 1997; Smith et al., 2007; Uemura et al., 1989; Wodarz et al., 2000, 1999). Therefore, it is a possibility that, in polarized NBs, asymmetric Mira localization at the basal pole might reflect spatial differences in aPKC activity. At the apical pole aPKC activity is high, driving Mira off the PM, but at the basal pole, at which aPKC activity is low, Mira continues to interact with the PM.

The situation appears to be more complex than these models suggest. Mira localizes uniformly on the PM in interphase (Sousa-Nunes et al., 2009). We have recently shown that, at the onset of mitosis, Mira is cleared from most of the PM by direct phosphorylation by aPKC (Hannaford et al., 2018). This suggests that the absence of Mira asymmetry in metaphase in *apkc* mutants (Rolls et al., 2003) may be a consequence of defective Mira clearance from the PM in prophase. It is possible that aPKC no longer contributes to Mira asymmetry in metaphase. Indeed, after nuclear envelope breakdown (NEB) actomyosin is required to keep Mira asymmetrically localized. However, disruption of the actin cytoskeleton after NEB also causes aPKC to become uniformly localized (Hannaford et al., 2018). Thus, the observed loss of Mira asymmetric localization upon actin network disruption might be indirectly caused by ectopic aPKC activity driving Mira off the PM at the basal NB pole. We therefore sought to directly address the contribution of aPKC to Mira localization specifically after NEB.

Temporal control over aPKC activity can be achieved by small molecule inhibitors. CRT90 has been used to inhibit aPKC function in the *Caenorhabditis elegans* zygote (Rodriguez et al., 2017) and in epithelia in *Drosophila* (Aguilar-Aragon et al., 2018). A disadvantage of kinase inhibitors is that they are often promiscuous and prone to off-target effects (Bain et al., 2003), which make the design of controls challenging. A solution to this problem is chemical genetics, relying on a kinase that is engineered such that it becomes sensitive to inhibitory ATP analogues, whereas the wild-type version of it does not (Bishop et al., 2000). This strategy has been used in yeast (Lopez et al., 2014) as well as mice (Kumar et al., 2015) and cultured cell lines (Wong et al., 2004).

Here, we report the generation of an analogue-sensitive (AS) allele of aPKC in *Drosophila*. We used this allele to shed light on the role of aPKC in patterning the localization of cell fate determinants in NBs. We find that aPKC activity appears to sharpen the pattern of Mira after NEB by removing Mira from the apical and lateral membrane.

## RESULTS

### Identification of an AS allele of *apkc* (*apkc^as4^*)

**Fig. 1.**
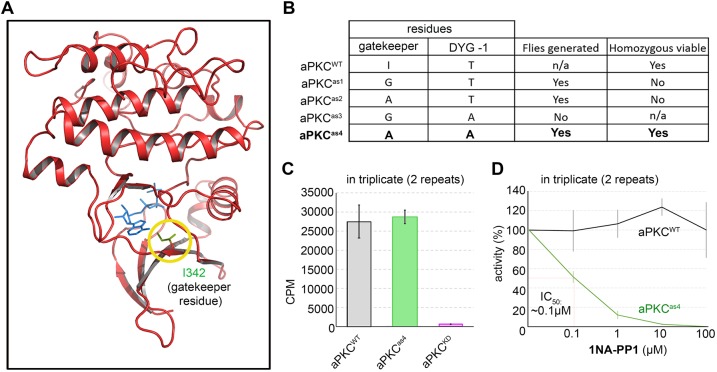
***In vitro* characterization of *apkc^as4^*.** (A) aPKC kinase domain homology model with the gatekeeper residue (yellow circle) I342 shown in green. The bound ATP analogue (blue sticks) is from a structural superposition with PKC beta (pdb code 3PFQ) (B) AS alleles of *apkc* generated and assessment of homozygous viability. (C,D) *In vitro* kinase assays. (C) aPKC^as4^ (I342A T405A) has comparable activity to aPKC^WT^ determined by the ability to phosphorylate a synthetic substrate. Mutation of D406 to alanine generates an inactive kinase (aPKC^KD^), validating the assay. (D) 1NA-PP1 specifically inhibits aPKC^as4^ but not the wild-type aPKC. We estimated an IC_50_ of ∼0.1 µM.

In order to assess the function of aPKC kinase activity with high specificity and temporal control in *Drosophila*, we sought to exploit chemical genetics and engineered an AS version of aPKC that can be inhibited by cell-permeable inhibitors (Bishop et al., 2000), for example 1NA-PP1. Based on sequence homology (Blethrow et al., 2004) and homology-based 3D structure modelling, we identified isoleucine 342 (I342) of *Drosophila* aPKC as the amino acid (termed gate keeper residue) that should be changed to construct AS alleles (Fig. 1A). We then used CRISPR (Gratz et al., 2013) to generate a range of potential *apkc as* alleles. Replacing I342 with glycine (*apkc^as1^*) or alanine (*apkc^as2^*) resulted in lethality when homozygous, which suggests that aPKC kinase activity is compromised in these mutants (Fig. 1B). To overcome this limitation, we introduced an additional mutation outside the adenosine-binding pocket in a subdomain that harbours the catalytic active site, the DFG motif (DYG in *Drosophila* aPKC), as the optimal AS allele configuration carries an alanine at the position immediately before the DFG motif (Blethrow et al., 2004). As *Drosophila* aPKC has a threonine at this position, we mutated it to alanine (T405A). Although we did not obtain any flies carrying the I342G and T405A (*apkc^as3^*) double mutation, the I342A and T405A double mutant (hereafter called *apkc^as4^*) resulted in homozygous viable and fertile flies (Fig. 1B). aPKC kinase activity of the protein encoded by *apkc^as4^* was consistently comparable with wild-type aPKC protein *in vitro*. A kinase dead version of aPKC (aPKC^KD^), in which we mutated the catalytic active site that harbours the DYG motif to AYG (Foukas et al., 2006; Okkenhaug et al., 2002), had little activity, validating the assay (Fig. 1C). Whereas wild-type aPKC (aPKC^WT^) did not respond to concentrations of up to 100 µM 1NA-PP1, aPKC^as4^ was readily inhibited by 1NA-PP1, with an estimated IC_50_ of ∼100 nM (Fig. 1D). Thus, aPKC^as4^ can be specifically inhibited by 1NA-PP1 *in vitro* using nanomolar concentrations.

### *apkc^as4^* phenocopies *apkc* loss-of-function in the presence of 1NA-PP1 *in vivo*

**Fig. 2.**
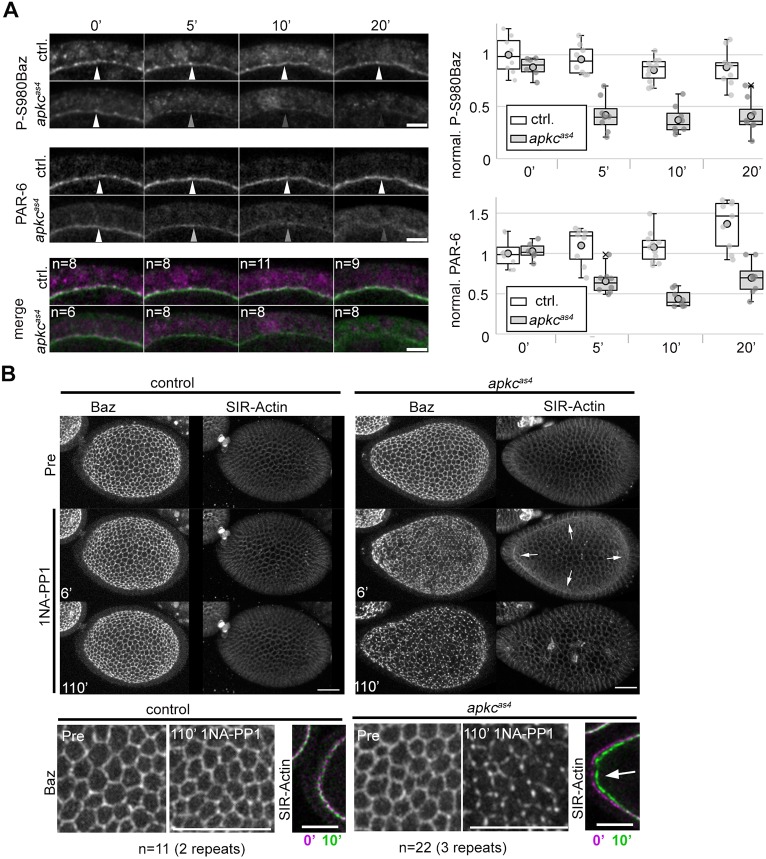
***In vivo* characterization of *apkc^as4^.*** (A) Follicle cells of the indicated condition were fixed and co-stained as indicated after 0′, 5′ 10′ or 20′ incubation with 20 µM 1NA-PP1. Inhibition of aPKC^as4^ causes strong reduction in apical signal of P-S980Baz and PAR-6 signal compared with controls at 5′ (apical, bottom panels). Arrowheads indicate differences in P-S980Baz and PAR-6 signal between controls and mutants. Box plots on right show quantification of P-S980Baz and PAR-6 signal normalized to the average value of the control at 0′. Median values (middle bars) and 25th and 75th percentile (boxes); whiskers indicate 1.5× the interquartile ranges; grey circles indicate individual data points. (B) Upper panels: maximum intensity projections of representative stills from living egg chambers (Movies 1 and 2.). After 10 µM 1NA-PP1 treatment, *apkc^as4^* mutants show defects in the organization of the apical domain and an increase in apical F-Actin (arrows) compared with controls. Lower panels: higher magnification of the apical domain and overlay of frames depicting the posterior cortex of egg chambers at 0′ (magenta) and 10′ (green) incubation with 1NA-PP1. Arrow indicates contraction of the posterior cortex in *apkc^as4^* mutant egg chambers. Scale bars: 5 µm in A; 10 µm in B (upper panels); 20 µm in B (lower panels).

We next determined whether aPKC^as4^ could also be inhibited *in vivo* and whether 1NA-PP1 would have any effect on wild-type tissues at the same concentration. In *Drosophila*, aPKC function has been well characterized in epithelia (Franz and Riechmann, 2010; Harris and Peifer, 2007; Hutterer et al., 2004; Rolls et al., 2003; Wodarz et al., 2000). aPKC phosphorylates Baz on a conserved residue, serine 980 (S980), for which specific antibodies (P-S980Baz) have been generated. Loss of *apkc* also alters the localization of PAR-6 in epithelial follicle cells (Krahn et al., 2009; Morais-de-Sá et al., 2010). We therefore used P-S980Baz and PAR-6 as a readout for aPKC activity. We incubated control and *apkc^as4^* mutant egg chambers with 1NA-PP1, fixed them at different time points and stained them to assess P-S980Baz and PAR-6 localization. In controls, both antibodies revealed the expected signal at the apical side of follicle cells even after 20 min in the presence of the inhibitor. Untreated *apkc^as4^* mutants also showed the expected apical signal of both. Upon addition of 1NA-PP1 to *apkc^as4^* mutants, P-S980Baz and PAR-6 levels at the apical side of mutant follicle cells declined after 5 min and reached levels found in the cytoplasm after 20 min (Fig. 2A). Thus, aPKC appears to be inhibited in *apkc^as4^* mutant follicle cells upon incubation with 1NA-PP1 within minutes with high specificity, as controls carrying wild-type aPKC do not respond to the inhibitor in this assay.

We further imaged living control or *apkc^as4^* mutant egg chambers expressing Baz::GFP and incubated with a SIR-Actin dye (Lukinavičius et al., 2014) to monitor the apical domain and the actin cytoskeleton, respectively. Before adding the inhibitor, imaging of both genetic backgrounds revealed the characteristic rotation of the egg chambers (Haigo and Bilder, 2011), which indicated that the preparations were healthy (Movies 1 and 2). Whereas the control continued to rotate and did not show any obvious response of the two reporters used, even after 2 h in the presence of the inhibitor (Movie 1), *apkc^as4^* mutants rapidly showed changes in the distribution of the SIR-Actin dye, which became enriched at the apical side of follicle cells and appeared to be paralleled by constriction of the egg chambers (Fig. 2B, Movie 2). These constrictions were apparent by changes in the geometry of adherens junctions that were labelled by Baz (the first changes in the distribution of Baz occurred 3.4±1.7 min after 1NA-PP1 addition, *n*=20). Upon aPKC inhibition, Baz::GFP accumulated at the apical medial domain before resolving into puncta (Fig. 2B), as described in fixed *apkc* mutant follicle cell clones and to some extent in egg chambers treated with CRT90 to inhibit aPKC activity (Aguilar-Aragon et al., 2018; Morais-de-Sá et al., 2010). These results based on live imaging are further consistent with a previous report that observed apical constriction in fixed follicle cells that express only Baz in which serine 980 is mutated to alanine and cannot be phosphorylated at this position by aPKC (Morais-de-Sá et al., 2010). Thus, aPKC kinase activity can be specifically and rapidly inhibited *in vivo* in *apkc^as4^* mutants, which recapitulates known phenotypes of *apk*c loss-of-function, whereas tissues with wild-type aPKC do not respond to the inhibitor in the assays used.

### Acute aPKC inhibition has different effects on polarity protein localization than *apkc* RNAi and can affect Baz positioning in larval NBs

**Fig. 3.**
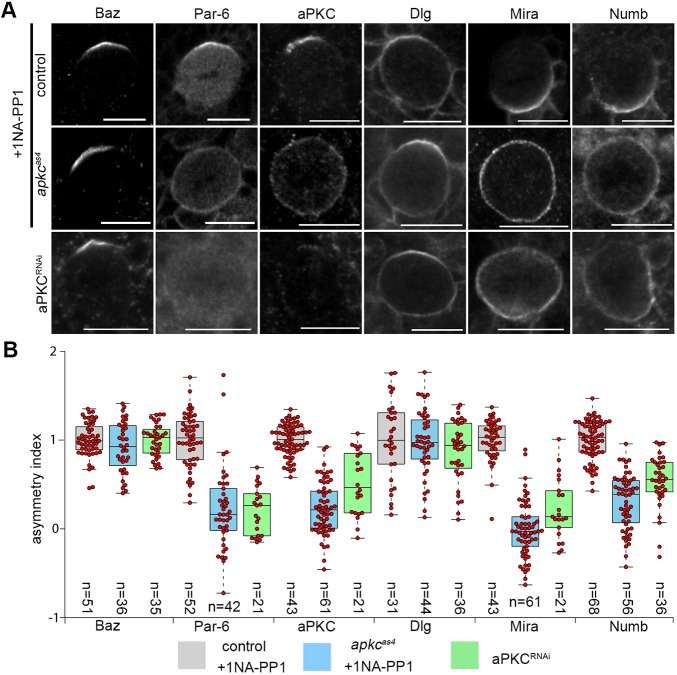
**The effect of acute aPKC inhibition on** NB **polarity compared with *apkc* RNAi.** (A) Representative images of mitotic larval NBs from control, *apkc^as4^* and *apkc* RNAi (driven by worniu-Gal4) brains. Living control and *apkc^as4^* brains were cultured in the presence of 10 µM 1NA-PP1 for 90 min to allow for at least one cell cycle and then stained to reveal the indicated polarity proteins. (B) Quantification of the ASI normalized to the wild-type immunofluorescence. Data pooled from three independent repeats. Median values (middle bars) and 25th and 75th percentile (boxes); whiskers indicate 1.5× the interquartile ranges; red circles indicate individual data points. Scale bars: 10 µm.

Having established that aPKC^as4^ can be inhibited *in vivo*, we tested the effect of acute aPKC inhibition on larval NB polarity, in which aPKC is known to play a role. We incubated living *w^1118^* and *apkc^as4^* brains in 1NA-PP1 for 90 min to cover at least one cell cycle before fixing and scoring for various polarity protein localizations in mitosis. In the presence of 1NA-PP1, *apkc^as4^* larval NBs displayed mis-localized Mira and Numb (Fig. 3A,B), recapitulating known *apkc* mutant phenotypes (Rolls et al., 2003; Smith et al., 2007), demonstrating aPKC^as4^ inhibition. Whereas Baz and Dlg formed apparently normal crescents upon aPKC inhibition and RNAi, aPKC and PAR-6 were predominantly localized uniformly on the mitotic NB cortex upon acute inhibition, but cytoplasmic or undetectable upon RNAi (Fig. 3A,B). Therefore, there is a difference in PAR-6 and aPKC localization between the two methods.

**Fig. 4.**
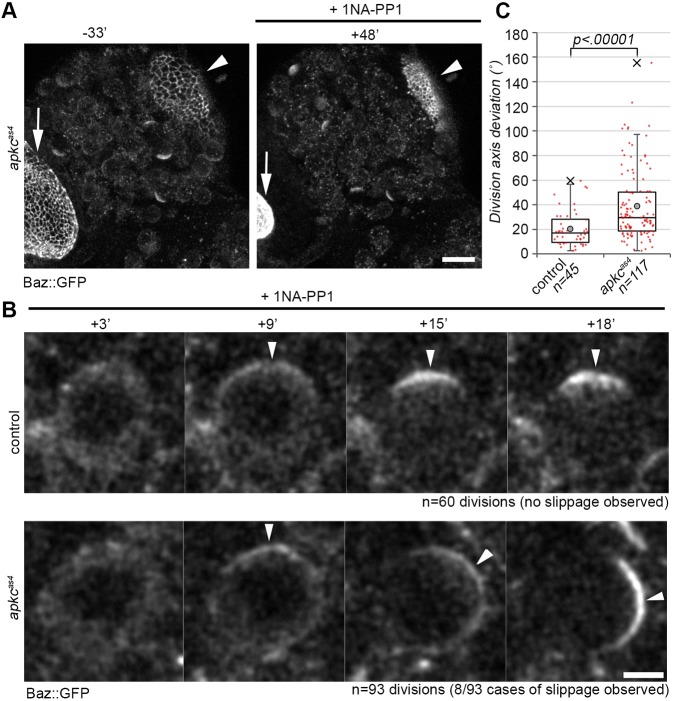
**Inhibition of aPKC kinase activity alters** NB **division orientation.** (A) Whole-mount brain preparation of *apkc^as4^* mutant flies that express Baz::GFP and Mira::mCherry (not shown) before (−33′) and after (+48′) addition of 10 µM 1NA-PP1 (Stills from Movie 3). Arrows indicate eye disc; arrowheads indicate neuroepithelium. (B) Higher magnification of a control and an *apkc^as4^* NB in the central brain entering mitosis and dividing in the presence of the inhibitor (Stills from Movies 4 and 5). Arrowheads trace the position of Baz::GFP. (C) Quantification of NB division orientation between consecutive cell cycles. Data pooled from three independent repeats of each condition. Median values (middle bars) and 25th and 75th percentile (boxes); whiskers indicate 1.5× the interquartile ranges; red dots indicate individual data points. Grey circles indicate the average and crosses indicate the maximum outlier. Scale bars: 20 µm in A; 5 µm in B.

Consistent with a previous report that used *apkc* loss-of-function alleles to determine the effect of loss of aPKC on Baz crescent formation (Rolls et al., 2003), we found that Baz crescents formed even in two consecutive mitoses of larval NBs under aPKC inhibition. Inhibition was evident in these samples by the rapid constriction of the apical domain in attached eye discs and neuroepithelia in the mutant (Fig. 4A, Movie 3), but constriction did not occur in the control (not shown). Therefore, acute aPKC inhibition in larval NBs does not prevent Baz crescent formation. However, we detected a few cases in which Baz positioning at the onset of mitosis appeared to be affected.

Normally, once Baz accumulates at the apical pole of larval NBs, it concentrates towards NEB and does not change its position thereafter (Fig. 4B, Movie 4). Interestingly, we found in ∼7% of *apkc^as4^* NBs (*n*=93) that went into mitosis and divided in the presence of the inhibitor, that Baz initially polarized at one pole of the NB cortex, but then changed position, which was not observed in the corresponding control NBs (Fig. 4B, Movie 5). Consistently, monitoring the orientation of NB division between consecutive cell cycles, that normally is stably maintained (Januschke and Gonzalez, 2010), revealed that aPKC inhibition induced a significant change in the orientation of the apico basal polarity axis between NB cycles (Fig. 4C). Embryonic NBs also show occasional defects in polarity axis orientation when mutant for *apkc* with a reduced ability to phosphorylate Baz (Kim et al., 2009). These results indicate that aPKC kinase activity may contribute to stabilizing the position of Baz in NBs at the onset of mitosis.

### Acute inhibition of aPKC does not affect Baz crescent formation in embryonic NBs

**Fig. 5.**
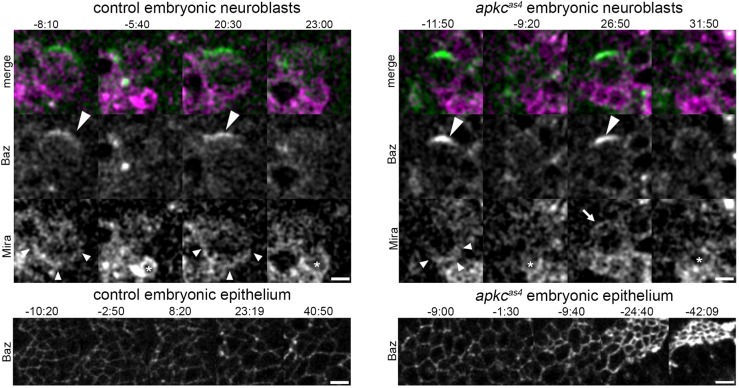
**Acute aPKC inhibition does not prevent Baz crescent formation in embryonic NBs.** Representative still images of cultures of stage 9 embryonic tissue of control (upper left panel) or *apkc^as4^* (upper right panel) mutants expressing Baz::GFP (green) and Mira::mCherry (magenta). Patches of embryonic tissues were cultured, and *z*-stacks imaged before and after the addition of 20 µM 1NA-PP1 (time point 0, not shown). Maximum projections of representative embryonic NBs are shown. Lower panels show projections of epithelial tissue in the cultures, allowing the constriction of the Baz::GFP labelled domain to be monitored, to demonstrate the effect of 1NA-PP1 on the mutant but not on the control. Large arrowheads indicate Baz crescents; small arrowheads indicate Mira crescents; arrow represents mis-localized Mira upon inhibition; asterisks show NB daughter cell produced during inhibitor treatment. Control sample size: *n*=22 divisions before NAPP1, *n*=31 divisions after NAPP1; mutant sample size: *n*=24 divisions before NAPP1, *n*=32 divisions after NAPP1. Four independent repeats of control and mutant conditions. Time indicated in mm:ss. Scale bars: 5 µm.

Our results and previous observations in larval NBs (Rolls et al., 2003) therefore differ from observations regarding the effect of loss-of-function alleles of aPKC on Baz localization in embryonic NBs. About one third of embryonic NBs that are derived from *apkc* mutant germ line clones lose the ability to form Baz crescents (Kim et al., 2009). Therefore, loss of Baz observed in *apkc* mutant embryonic NBs might reflect indirect or cumulative effects of loss of aPKC protein and may not occur upon acute aPKC inhibition in embryonic NBs. To test this, we made cultures of patches of stage 9 embryonic tissues that harboured epithelial cells as well as NBs from control (Movie 6) or homozygous mutant *apkc^as4^* embryos (Movie 7) that expressed Baz::GFP and Mira::mCherry. We then treated the cultures with 1NA-PP1 and monitored the localization of Baz in both backgrounds. The effect of acute aPKC inhibition was evident in the mutant (Movie 8), but not in the control (Movie 9), by the ensuing apical constriction of epithelial cells [epithelia started contracting 5.2±1 min (*n*=15 from four independent experiments) after 1NA-PP1 addition] as well as by the loss of Mira asymmetry in mitotic embryonic NBs. Baz crescents in mitotic embryonic NBs were readily detectable in the control as well as in the mutant before and after the addition of 1NA-PP1 (Fig. 5). These results show that acute inhibition of aPKC allows for Baz crescent formation in embryonic NBs. The time resolution and conditions of our embryonic culture protocol did not allow us to faithfully determine whether acute inhibition of aPKC also causes slippage of Baz position as observed in larval NBs, which therefore remains to be determined.

### aPKC activity sharpens the pattern of Mira asymmetry after NEB of larval NBs

Having established that aPKC can be inhibited in fly tissues with high specificity and temporal control, we next wanted to dissect the specific requirements of aPKC kinase activity on fate determinant localization at different time points in the cell cycle of larval NBs, which cannot be addressed by RNAi or clonal analysis of *apkc* loss-of-function.

During interphase, Mira uses its membrane interacting basic and hydrophobic (BH) motif to localize uniformly at the cortex. At the onset of mitosis, serine 96 (S96) of Mira is phosphorylated by aPKC, which inhibits membrane interaction and clears Mira into the cytoplasm (Bailey and Prehoda, 2015; Hannaford et al., 2018). After NEB, Mira regains the ability to localize in a basal crescent, which requires the BH motif and actomyosin dependent processes (Hannaford et al., 2018). The contribution of aPKC kinase activity to the asymmetric localization of Mira after NEB is unknown. Therefore, we used the temporal control that our chemical genetics approach offers to address this issue.

**Fig. 6.**
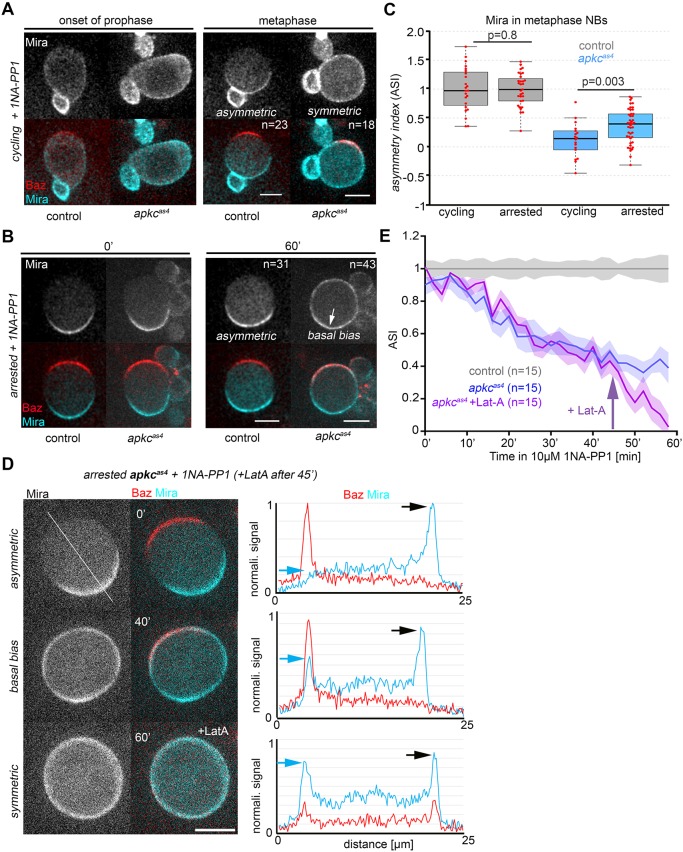
**aPKC activity sharpens the asymmetric pattern of Mira after NEB in larval NBs.** (A,B) Larval NBs in primary cell culture expressing Baz::GFP (red) and Mira::mCherry (blue). (A) Control and *apkc^as4^* NBs cycling into colcemid arrest before (onset of prophase, 0′) and after (metaphase, 30′) incubation with 10 µM 1NA-PP1. (B) Colcemid-arrested NBs before (0′) and after (60′) incubation with 10 µM 1NA-PP1. After 1 hour in the presence of the inhibitor (60′) Mira decorates the apical and lateral cortex, but continues to localize with a basal bias (arrow). (C) Quantification of Mira ASI normalized to the mean of the control (cycling or arrested) from experiments shown in A and B. Quantification was performed 60 min after the addition of 1NA-PP1. Median values (middle bars) and 25th and 75th percentile (boxes); whiskers indicate 1.5× the interquartile ranges; red circles indicate individual data points. Significance determined by *t*-test (two tailed, equal variance). Cycling control sample size: 23 from three independent repeats; arrested control sample size: 31 from three independent experiments; cycling mutant sample size: 18 from three independent experiments; arrested mutant sample size: 43 from three independent experiments. (D) Representative images from Movie 10. *apkc^as4^* NBs expressing Baz::GFP and Mira::mCherry were arrested with colcemid to allow polarization, then 10 µM 1NA-PP1 was added. After 45 min, 5 µM Lat-A was added to disrupt the actin network. Panels on the right show corresponding fluorescence profiles of Baz and Mira at the apical (blue arrow) and basal (black arrow) cortex at 0′, 40′, 60′. Fluorescence was measured using a 20px wide bar running in the apical to basal direction (orientation of bar indicated by white line, top left panel) on sum projections covering 4.8 µm (six *z*-planes). (E) Quantification of changes of Mira ASI over time normalized to the control at each time point from the experiments presented in B and of colcemid arrested *apkc^as4^* NBs that were first treated with 10 µM 1NA-PP1 and after 45 min, in addition incubated with 5 µM Lat-A (Movie 14). Control, two independent experiments; the other two conditions each three independent experiments. Shaded areas indicate s.e.m. Scale bars: 10 µm.

We first confirmed that acute aPKC inhibition interferes with the clearing of Mira from the cortex into the cytoplasm at the onset of mitosis, an expected effect of interfering with aPKC (Hannaford et al., 2018). This was indeed the case in inhibitor-treated *apkc^as4^* NBs, but not in treated controls (Fig. 6A, cycling, Movies 10 and 11). Remarkably, measuring the time from 1NAPP-1 addition to NEB, incubation for 9.9±0.6 min (*n*=4) with 10 µM 1NA-PP1 was sufficient to block Mira clearing, which resulted in uniform cortical Mira in mitosis (Fig. 6A, cycling). Thus, 1NA-PP1 at a concentration of 10 µM appears to rapidly and efficiently block aPKC^as4^ activity, mimicking the phenotype of loss of *apkc* on Mira or the behaviour of MiraS96A, which cannot be phosphorylated by aPKC at this residue, in cycling NBs (Hannaford et al., 2018).

To test whether aPKC activity contributes to asymmetric Mira localization after NEB, we arrested NBs with colcemid to activate the spindle assembly checkpoint, which allows NB polarity to develop normally (Broadus and Doe, 1997; Januschke and Gonzalez, 2010), and then added 10 µM 1NA-PP1. This treatment had no effect on controls in this assay (Fig. 6B, arrested, Movie 12). In arrested *apkc^as4^* NBs, Mira accumulated at the apical and lateral cortex, but it continued to localize with a detectable basal bias even after extended incubation with 1NA-PP1 (Fig. 6B, arrested, Movie 13). Indeed, there was a significant difference when comparing the asymmetry index (ASI) for Mira between 1NA-PP1-treated NBs that cycled into mitosis with those that were already arrested (Fig. 6C). Longer incubation of colcemid-arrested *apkc^as4^* NBs with the inhibitor eventually led to uniform cortical Mira (*not shown*). Nonetheless, in *apkc^as4^* NBs that were treated for 45 min with 1NA-PP1, the basal bias of Mira was lost upon disruption of the actin cytoskeleton by latrunculin A (Lat-A, Fig. 6D,E, Movie 14). These results reveal that aPKC clearly contributes to Mira asymmetry after NEB, by keeping it off the apical and lateral membrane. This activity appears rather to sharpen the pattern of Mira in mitotic NBs, as an actomyosin-dependent basal bias remains detectable.

## DISCUSSION

Here, we report the generation of an AS allele of *Drosophila* aPKC and demonstrate that it can be specifically inhibited *in vitro* and *in vivo*. Homozygous mutant *apkc^as4^* flies are viable and fertile, allowing the study of the effect of acute aPKC in all drug-accessible tissues with high specificity, while providing the best possible control, wild-type tissue that can be inhibitor treated (Figs 1 and 2).

When validating this new allele, we examined the effect of aPKC inhibition on cell polarity in various cell types. As observed in the *C. elegans* zygote (Rodriguez et al., 2017), depletion of aPKC by RNAi and acute inhibition of aPKC had different effects in *Drosophila* larval NBs: *apkc* RNAi resulted in cytoplasmic PAR-6, whereas aPKC inhibition resulted in the redistribution of PAR-6 and aPKC to the entire cortex. Neither *apkc* RNAi nor inhibition prevented Baz crescent formation (Fig. 3). Thus, it appears that in both the *C. elegans* zygote and larval NBs, acute aPKC inhibition decouples the localizations of Baz and aPKC.

This is different from mammalian and *Drosophila* epithelial cells, in which Par-3 and aPKC coupling is disrupted by the phosphorylation of a serine within the aPKC-binding site of Baz by aPKC. Baz carrying an alanine substitution for the corresponding conserved serine residue in *Drosophila* (Baz^S980A^) immunoprecipitates more aPKC than wild-type Baz in ovarian extracts showing that, without aPKC phosphorylation of this residue, aPKC and Baz interaction is stabilized in those systems (Morais-de-Sá et al., 2010; Nagai-Tamai et al., 2002). Expressing Baz^S980A^ in a *baz* mutant background in *Drosophila* affected aPKC localization in epithelia, but phosphorylation at this site does not seem to be required for the polarity of embryonic NBs (Morais-de-Sá et al., 2010). In contrast to this data, we find that acute aPKC inhibition in larval NBs appears to uncouple aPKC from Baz localization (Fig. 3). Baz/aPKC coupling in NBs therefore may depend upon a different aPKC phosphorylation event.

We further find that aPKC activity may regulate some aspects of the localization of Baz itself. This was already suggested by the fact that about one third of embryonic NBs that are derived from *apkc* mutant germline clones failed to asymmetrically localize Baz (Kim et al., 2009). However, aPKC inhibition did not prevent Baz crescent formation in embryonic or larval NBs (Figs 4 and 5), which appears to illustrate that absence of aPKC protein and inhibition of its kinase activity are not strictly equivalent. Nevertheless, although Baz crescents always formed without aPKC activity in larval NBs, in a small number of cases their positions slipped from their original place (Fig. 4). This may point to a direct role for aPKC kinase activity in stabilizing the position of Baz. However, more indirect causes for Baz crescent slippages cannot be ruled out. For example, constricting attached discs and/or neuroepithelia of the optic lobes may alter mechanical properties of the brain upon aPKC inhibition. Indeed, in *C. elegans,* Baz polarization was proposed to be force sensitive (Wang et al., 2017), which may also be a possibility in larval NBs (Loyer and Januschke, 2018).

We were prompted to generate an AS allele of aPKC by the need for temporal control of inhibition to further investigate aPKC function in controlling Mira localization in *Drosophila* NBs. Blocking aPKC activity all along the cell cycle of NBs recapitulated the expected Mira mis-localization phenotype (Fig. 6) (Hannaford et al., 2018; Rolls et al., 2003; Wodarz et al., 2000), and blocking aPKC activity after NEB resulted in a redistribution of Mira from the basal pole to the rest of the cortex (Fig. 6). Therefore, aPKC appears to negatively regulate Mira cortical association during NB polarity establishment and its maintenance.

However, the redistribution of Mira upon aPKC inhibition in colcemid-arrested NBs was too slow to cause a complete loss of asymmetry in polarized NBs within the time frame of a normal mitosis, which on average lasts only for ∼15 min (Fig. 4). This could stem from inefficient inhibition of aPKC in arrested *apkc^as4^* NBs. However, this appears to be unlikely as the same concentration of 1NA-PP1 is sufficient to block Mira clearing from the interphase membrane at the onset of mitosis in NBs treated for only ∼10 min, and that cycled into colcemid-induced mitotic arrest resulted in symmetric Mira localization (Fig. 6A). Our results appear to strengthen the view that actomyosin-dependent processes contribute to maintaining Mira asymmetry after NEB in larval NBs. We find that basally localized Mira remains stabilized by the actin network even under acute inhibition of aPKC (Fig. 6A). Thus, consistent with our recent findings (Hannaford et al., 2018), direct phosphorylation of Mira by aPKC to keep it off the mitotic NB PM appears to be unlikely to be the sole mechanism that maintains Mira asymmetry. Asymmetric Mira localization in mitosis may therefore rely on an underlying actomyosin-dependent patterning mechanism.

An interesting line of future investigation could be to test the involvement of aPKC in such a mechanism. It is possible that the phosphorylation status of Mira may regulate its ability to engage with this actin-dependent maintenance mechanism: aPKC activity would not only prevent the binding of Mira to the PM, but also promote its actin-dependent maintenance at the basal pole. This would be consistent with recent findings that both actomyosin-dependent processes and the aPKC-regulated PM association domain of Mira are required for asymmetric Mira localization in larval NBs (Hannaford et al., 2018). aPKC may also directly regulate aspects of the actomyosin-dependent patterning of NBs. In follicle epithelial cells, acute aPKC inhibition triggers contractions (Fig. 2) that are likely driven by apical constrictions, also observed in this tissue in cells that express the phosphomutant Baz^S980A^ (Morais-de-Sá et al., 2010). Therefore, aPKC kinase activity may counteract myosin activity in NBs, as it does in follicle cells and in other contexts (David et al., 2013; Kishikawa et al., 2008), for example through regulation of ROCK (Ishiuchi and Takeichi, 2011; Röper, 2012). Asymmetries in the actomyosin network can be detected during NB mitosis (Barros et al., 2003) and operate in the control of size-asymmetry between NBs and their daughter cells (Roubinet et al., 2017). How these asymmetries arise remains unclear, but it is a possibility that they are regulated by aPKC and contribute to patterned fate determinant localization.

In summary, we have demonstrated that *Drosophila* is amenable to chemical genetic approaches to assay kinase function. In this example, through temporal inhibition of aPKC activity we have started to address its respective contributions to the establishment and maintenance of asymmetric cell fate determinant localization in NBs. It would be interesting to further this approach to dissect the precise contributions of other kinases that are implicated in asymmetric cell division.

## MATERIALS AND METHODS

### Fly lines

Flies were reared on Nutri-fly food (Bloomington formulation, Genesee Scientific) at 25°C. The lines used were: *Baz::GFP* (Buszczak et al., 2007), *UAS-aPKCRNAi*: P{y[+t7.7] v[+t1.8]=TRiP.HMS01320}attP2 (Bloomington Drosophila Stock Center, #34332), *miranda::mcherry* (Ramat et al., 2017). Worniu-Gal4 was provided by C. Doe (Institute of Neuroscience, Institute of Molecular Biology, Howard Hughes Medical Institute, University of Oregon, USA) (Albertson et al., 2004). For the generation of *apkc^as4^* we used 5′ CGAGGTCGACGGTATCGATACTAGCCTGCCGTAAGCATG and 5′ GAAAGCACGCTGGAACGGAAGTTTGTTTGTATATTC to amplify the relevant genomic region from BAC CH321 09L15 and cloned it into pBlueskript SK+. We subsequently used site-directed mutagenesis to introduce the I342A and T405A mutations, an *Eco*R1 site for diagnosis and to silence the gRNA restriction sites. We then used PCR on BAC CH321 09L15 with 5′ CGAGGTCGACGGTATCGATACTAGCCTGCCGTAAGCATG and 5′ GAAAGCACGCTGGAACGGAAGTTTGTTTGTATATTC to amplify the 5′ homology arm and with 5′ TCCCGATCAGGTAACCATATTCATTTATTTTGAGAAATTCTAATTTG and 5′ CCGGGCTGCAGGAATTCGATGGGGGCTTATCTCATGCAAG to amplify the 3′ homology arm, and used Gibson cloning to assemble the sequences into pBluescript SK+. This was co-injected with the guide RNA 5′ TATATAGGAAAGATATCCGGGTGAACTTCGGTCATCGAGTTTGTGCGCGGGTTTTAGAGCTAGAAATAGCAAG that was cloned into pCFD3 (Port et al., 2014) into y[1] M{vas-Cas9}ZH-2A w[1118]/FM7c (Bloomington Drosophila Stock Center, #51323). Injected flies were crossed to relevant balancer stocks and flies were genotyped using non-lethal PCR (Carvalho et al., 2009) on wings with 5′ ACGACTTCGAGCTGATAC and 5′ GTACCGCAGAATGTGGAGGT. The PCR product was analyzed with *Eco*R1 (introduced into the template) and *Lgu*I (endogenous, lost in the template) digestion and the positive flies were sequenced to validate the successful engineering of the aPKC locus. A similar strategy was used to mutate I342 to glycine (I342G: as1) or alanine (I342A: as2) and the combined mutation I342G, T405A (as3).

### Cloning

The coding sequence of aPKC-RA was amplified by PCR using primers which contained 5′ *Eco*R1 and 3′ *Not*I restriction sites (Template plasmid, a gift from A. Wodarz, Institute for Anatomy, University of Cologne, Germany). The amplicon was then digested using *Eco*R1 and *Not*1 and inserted into pCMV5-Flag1 (N-terminal FLAG tag, MRC Protein Phosphorylation & Ubiquitylation Unit). AS aPKC mutants were then generated by gene synthesis and subcloned into this vector by *Bam*H1 and *Not*1 restriction digest. To generate FLAG-aPKC^KD^, aPKC was subcloned into pBluescript and mutated by Gibson assembly, using primers which contained mutations that changed the catalytical active site (DYG in Drosophila aPKC) to AYG (Foukas et al., 2006). aPKC^KD^ was then cloned back into pCMV5-Flag1 using *Eco*R1 and *Not*1 restriction digest.

### Kinase assay

HEK293 were purchased from ATCC and cultured in Dulbecco's modified Eagle medium (Glutamax, Gibco) which was supplemented with 10% foetal calf serum (FCS), 100 U/ml penicillin and 100 µg/ml streptomycin at 37°C in a humidified atmosphere with 5% CO_2_. Cells were regularly tested for mycoplasma contamination and confirmed as negative for experimental analysis. Transient transfections were performed on 70% confluent cells using plasmid DNA and polyethylenimine PEI Max (0.1% w/v) (Polysciences) in a 1:3 ratio. Cells were lysed 48 h after transfection in ice-cold lysis buffer containing 50 mM Tris/HCl (pH 7.5), 1% (v/v) Triton X-100, 1 mM EGTA, 1 mM sodium orthovanadate, 50 mM NaF, 10 mM 2-glycerophosphate, 5 mM sodium pyrophosphate, 270 mM sucrose and cOmplete EDTA-free Protease Inhibitor Cocktail (Roche). Lysates were clarified by centrifugation at 20,800 ***g*** for 10 min at 4°C and the protein content of supernatants were quantified using Bradford assay. FLAG-aPKC overexpression was verified by western blotting analysis using an anti-FLAG M2 monoclonal antibody (Sigma-Aldrich, F3165, 1:2000). The IC50 of 1NA-PP1 (Calbiochem, #529579, MW 317) was determined in an *in vitro* peptide substrate phosphorylation assay using immunoprecipitated wild-type and mutant variants of aPKC. For this purpose, FLAG-tagged wild-type kinase-dead and AS aPKC were transiently overexpressed in HEK293 cells (as described above) and aPKC immunoprecipitated from cell extracts using anti-FLAG M2-agarose (Sigma-Aldrich) for 1 h at 4°C. A control was also included in which HEK293 cells were transfected with FLAG-empty vector. Immunoprecipitates were then washed 3× with lysis buffer that was supplemented with 300 mM NaCl, and 2× with 50 mM Tris/HCl (pH 7.5). Peptide kinase assays were set up with aPKC that was immunoprecipitated from 30 μg of cell extract in 50 mM Tris/HCl (pH 7.5), 0.1 mM EGTA, 10 mM MgCl_2_, 0.1 mM [γ-^32^P]ATP (∼300-500 CPM/pmol, PerkinElmer) and 120 μM peptide substrate (ERMRPRKRQGSVRRRV) (Balendran et al., 2000) in the presence of the indicated concentration of inhibitor. After incubation for 30 min at 30°C with shaking, reactions were terminated by applying the reaction mixture on to P81 phosphocellulose papers and immersing in 50 mM orthophosphoric acid. After extensive washing in 50 mM orthophosphoric acid, the radioactivity in the reaction products was quantified using Cerenkov counting.

### Live imaging

For isolated NBs, brains were dissected in collagenase buffer and incubated in collagenase for 20 min. Brains were then transferred to a drop of fibrinogen (0.2 mg ml^−1^) on a 25 mm glass-bottom dish before being manually dissociated with needles. The fibrinogen was clotted using thrombin (100 U ml^−1^, Sigma-Aldrich, T7513). After 10 min, Schneider's medium supplemented with FCS, Fly Extract (Drosophila Genomics Resource Center, 1645670) and insulin (Sigma-Aldrich, I0516) was pipetted on top (Pampalona et al., 2015). Live imaging of NBs was performed using a Yokogawa CSU-X1 spinning disc unit mounted on an Olympus IX81 microscope using a 100× Planapo 1.45 NA Olympus objective. Maximum intensity projections of six optical planes 0.8 µm apart around the equator of the cells are shown. Data was processed (3D gaussian blur: 0.8/0.8/0.8) and analyzed using FIJI (Schindelin et al., 2012).

For egg chambers, ovaries were dissected from 24 h old female flies and placed into a drop of glucose- (1 g l^−1^) and insulin- (0.2 g l^−1^) supplemented Schneider's medium (SLS-04-351Q, Sigma-Aldrich) in a 25 mm glass-bottom dish (World Precision Instruments) and dissected into individual ovarioles, which were clotted in fibrinogen as indicated above. They were then imaged on a SP8 confocal microscope (Leica) equipped with a 63× NA 1.2 water immersion objective.

For embryonic NB cultures, for each condition, six embryos were manually dechorionated 4 h after egg laying, rinsed in 70% ethanol and distilled water, and manually dissociated, which resulted in patches of tissue containing epithelial cells and NBs in a drop of supplemented Schneider's medium containing bovine fibrinogen (Sigma-Aldrich, 0.2 mg ml^−1^). NBs were identified by their ability to form Baz and Mira crescents and to divide size-wise asymmetrically. The dissociated embryonic tissues were then transferred to a poly-lysin-coated glass-bottom dish (World Precision Instruments) and left for 20 min. The fibrinogen was clotted using thrombin (100 U ml^−1^, Sigma-Aldrich, T7513) for 10 min and supplemented Schneider's medium was pipetted on top. Embryonic NBs were imaged on a spinning disk confocal microscope (Yokogawa CSU-X1) using a 100× oil objective (NA1.45) or an SP8 confocal microscope (Leica) equipped with a 63× NA 1.2 water immersion objective. 1-NA-PP1 was added to a final concentration of 20 µM during the imaging. The only cells considered for our analysis as NBs were cells that expressed both Baz::GFP and Mira::mCherry, performed an asymmetric division before the addition of NA-PP1 and divided again within the next hour.

### Colcemid treatment

NB cultures were treated with 50 µM colcemid (Calbiochem, dissolved in 100% dimethyl sulfoxide) as described previously (Januschke and Gonzalez, 2010).

### Immunofluorescence

Brains and egg chambers were fixed for 20 min in 4% formaldehyde (Sigma-Aldrich) in PBS. Primary antibodies were incubated in PBST (PBS containing 0.1% Triton X-100) overnight. Primary antibodies used were as follows: rabbit anti-PKCζ (Santa Cruz, sc-17781, 1:1000), guinea pig anti-Numb (1:1000, provided by J. Skeath, Department of Genetics, Washington University in St. Louis, USA; O'Connor-Giles and Skeath, 2003), rabbit anti-Miranda (1:1000, provided by C. Gonzalez, Institute for Research in Biomedicine, Barcelona, Spain; Mollinari et al., 2002), rabbit anti P-S980-Bazooka,(1:1000, provided by A. Wodarz; Krahn et al., 2009), guinea pig anti-Par6 (1:500, provided A. Wodarz; Kim et al., 2009), mouse anti-Dlg (Developmental Studies Hybridoma Bank, 4F1, 1:500). Secondary antibodies used were (Life Technologies, all used at 1:2000): goat anti-rabbit Alexa 488 (A11034), goat anti-guinea pig Alexa 647 (A21450), goat anti-mouse Alexa 647 (A21325), goat anti-mouse Alexa 488 (A11029), donkey anti-rabbit Alexa 594 (A21207). Images were taken using a Leica-SP8 confocal microscope with a 63× water objective (NA 1.2).

### Data analysis

Intensity measurements were measured by performing a rotating line scan on the apical or basal cell cortex to define the peak intensity. ASI was defined as described by Rodriguez et al., 2017 using the following equation:

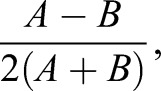


where A and B are apical and basal, respectively. For all experiments, the intensity was normalized to the wild-type control in the presence of 1NA-PP1.

### aPKC modelling

The aPKC kinase domain (Phe^264^-Phe^532^) homology model was generated using the intensive mode of the Phyre2 fold recognition server (Kelley et al., 2015). A structural alignment with CDK1 (pdb code 4YC3; Brown et al., 2015) was used to assign I^342^ as gatekeeper residue. Figures were prepared using PyMOL (Schrödinger).

### Spindle orientation measurements

The orientation of the division axis of NBs was measured by 3D vectors defined by the 3D coordinates of the apical (based on Baz::GFP) and the basal (based on Mira::mCherry) pole at metaphase as in Loyer and Januschke (2018).

The angle (α) between two 3D vectors was calculated using the formula:

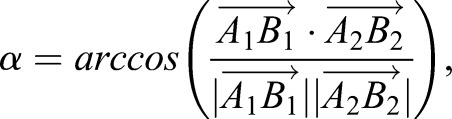


in which the dot product is:

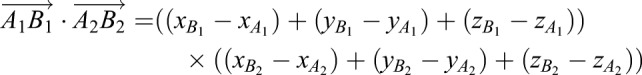
and the magnitude of any vector is:

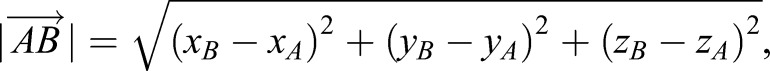


with 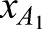 being for example the *x*-coordinate of the apical pole during the first division, and 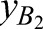
being for example the *y*-coordinate of the basal pole during the second division.

## Supplementary Material

Supplementary information
