# Supplementary material for: A chemical-genetics approach to study the role of atypical Protein Kinase C in Drosophila
Source: Development. 2019 Jan 29;146(2):dev170589. doi: 10.1242/dev.170589 (PMC6361133; doi:10.1242/dev.170589)
Supplement: Supplementary information [file develop-146-170589-s1.pdf]

Movies

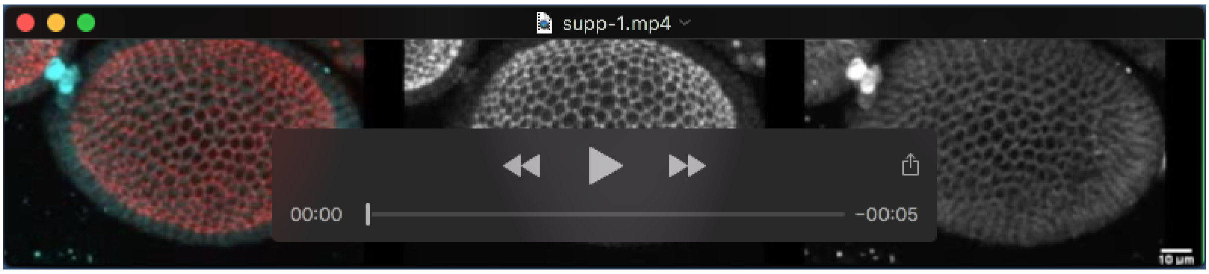

Movie 1 – *Baz localization is not affected by 1NA-PP1 in control follicle cells*. Control egg chambers expressing Baz::GFP (middle panel, red in merge) and stained with SIR-Actin (left panel, cyan in merge) prior and after addition of 10μM 1NA-PP1. Z stacks collected every 2min, movie displayed at 12 frames per second. 0′ marks the addition of 10μM 1NA-PP1. Time hh:mm.

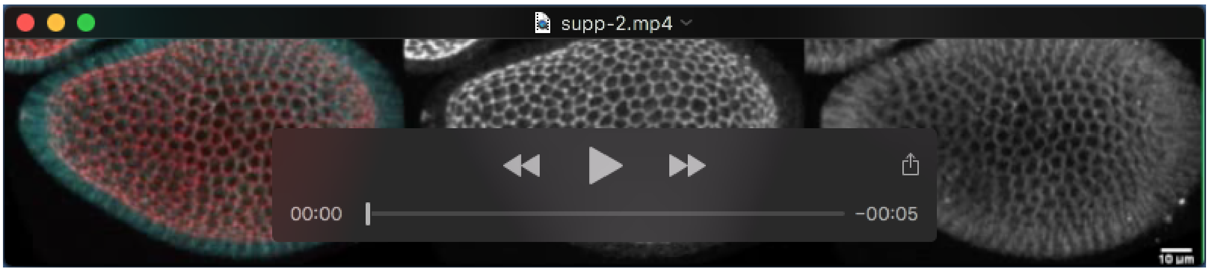

Movie 2 – *Baz localization is rapidly affected upon acute inhibition of aPKC in follicle cells*. Homozygous *apkc*^{as4} mutant egg chambers expressing Baz::GFP (middle panel, red in merge) and stained with SIR-Actin (left panel, cyan in merge) prior and after addition of 10μM 1NA-PP1. Z stacks collected every 2min, movie displayed at 12 frames per second. 0′ marks the addition of 10μM 1NA-PP1. Time hh:mm.

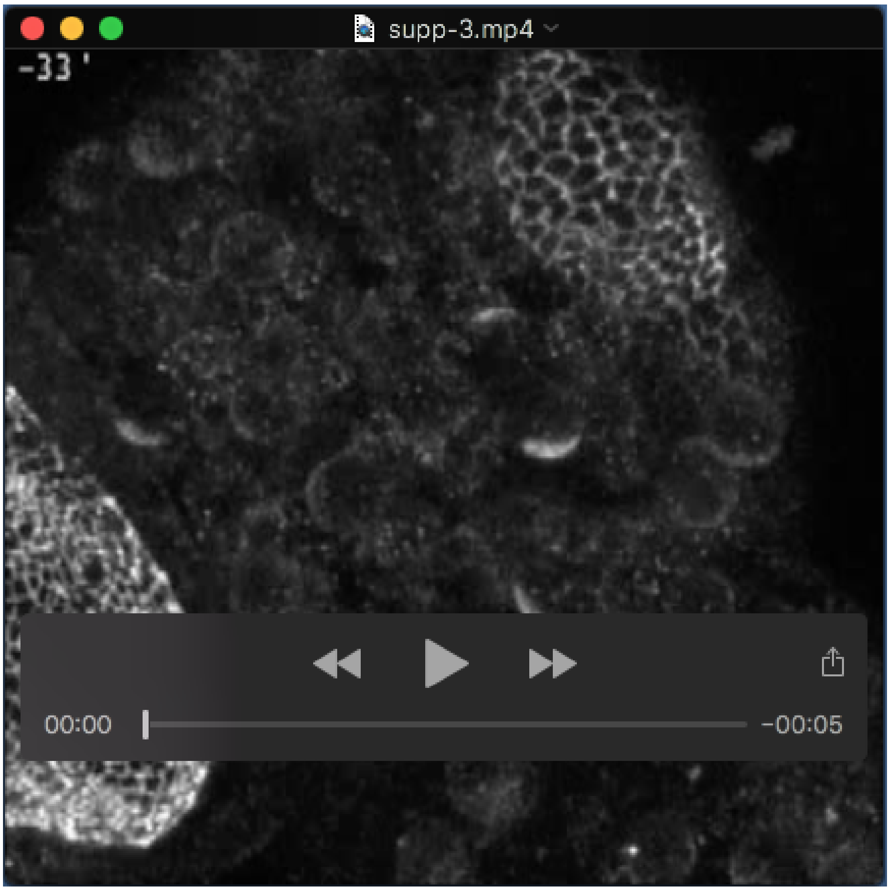

Movie 3 – *Acute inhibition of aPKC leads to apical constriction of eye discs and the neuroepithelium in whole mount brains*. Whole mount *apkc*^{as4} mutant brain expressing Baz::GFP and Mira::mCherry (not shown) prior and after addition of 10µM 1NA-PP1. Z stacks collected every 3min, movie displayed at 7 frames per second. 0′ marks the addition of 10µM 1NA-PP1. Time hh:mm.

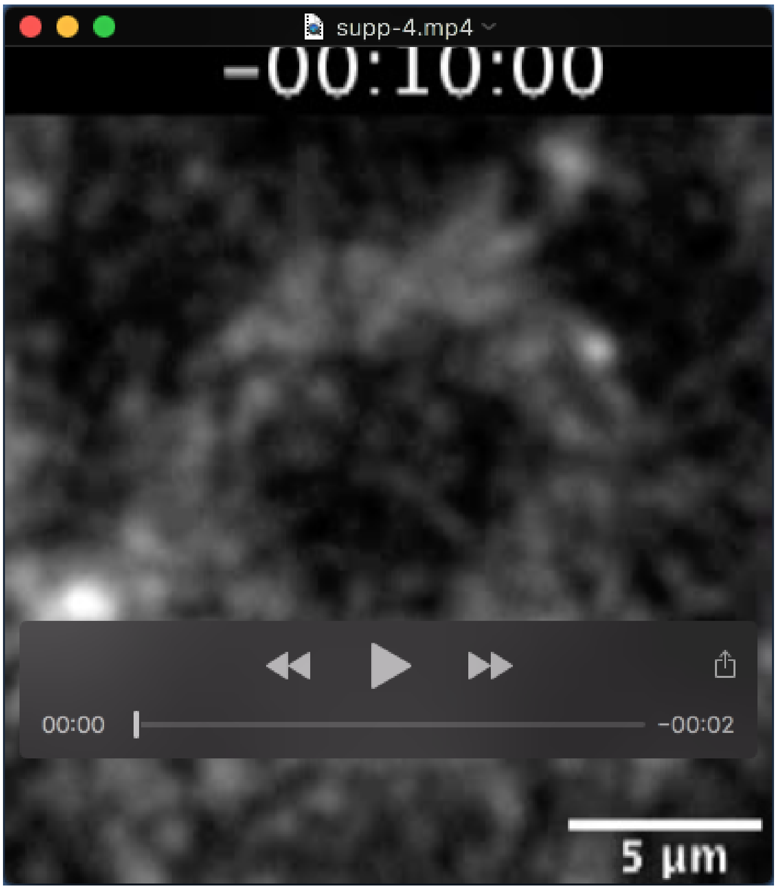

Movie 4 – *Baz localizes normally in control larval NBs upon 1NA-PP1 addition*. Higher magnification of a NBs from a control whole mount brain expressing Baz::GFP and Mira::mCherry (not shown) after addition of 10μM 1NA-PP1. Arrowhead traces center of Baz position. Z stacks collected every 3min, movie displayed at 7 frames per second. 0′ marks the addition of 10μM 1NA-PP1. Time hh:mm.

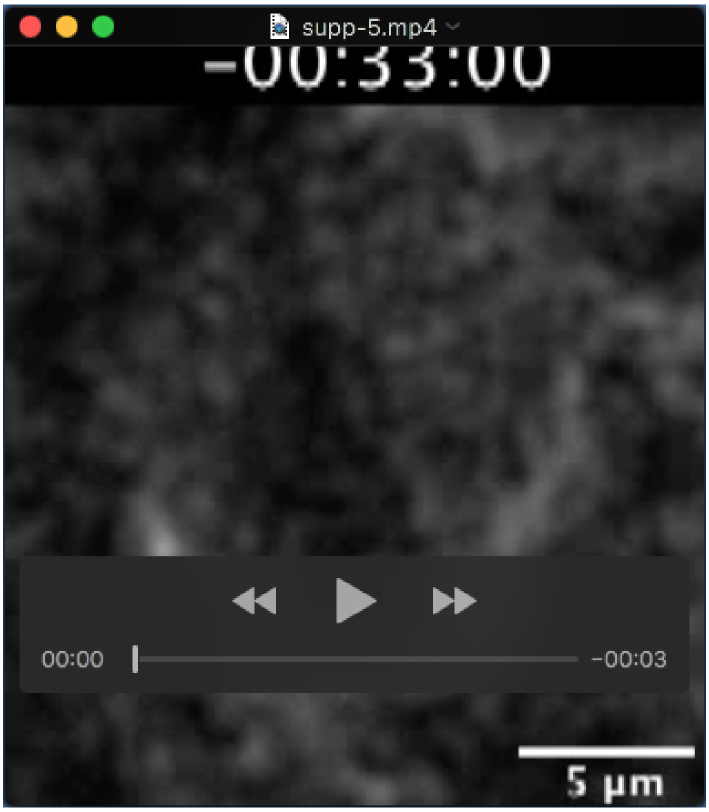

Movie 5 – *Baz localization is not always maintained at the onset of mitosis upon acute inhibition of aPKC in larval NBs*. Higher magnification of a NBs from an *apkc*^{as4} mutant whole mount brain expressing Baz::GFP and Mira::mCherry (not shown) after addition of 10µM 1NA-PP1. Z stacks collected every 3min, movie displayed at 7 frames per second. 0' marks the addition of 10µM 1NA-PP1. Time hh:mm.

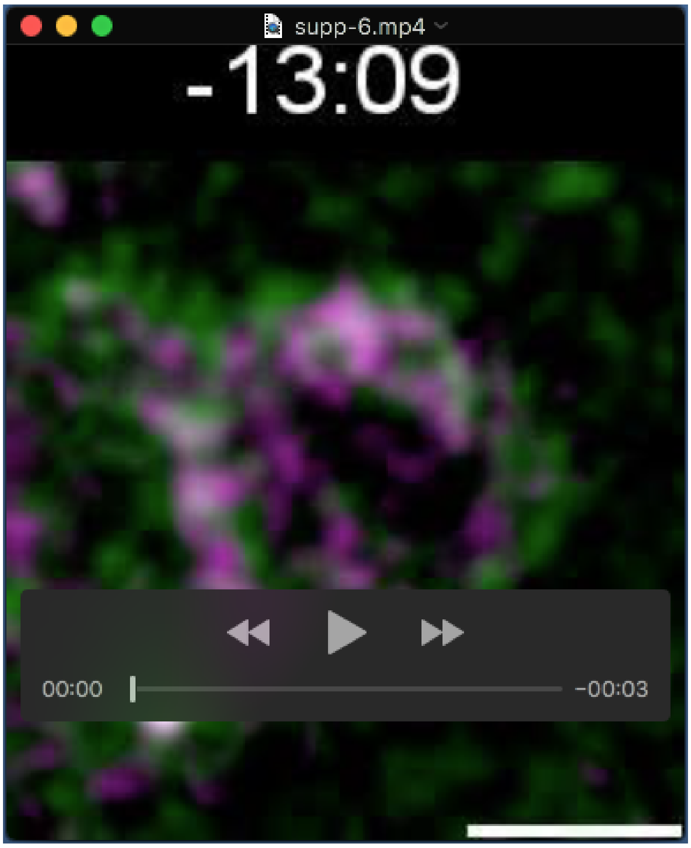

Movie 6 – *Control embryonic NB polarizes Baz and Mira normally before and after addition of 1NA-PP1*. Example of a control embryonic NB expressing Baz::GFP (green) and Mira::mCherry (magenta). Z stack collected every 2min30sec, movie displayed at 3 frames per sec. Time: mm:ss. 00:00 marks the addition of 20 μM 1NA-PP1. Scale bar: 5 μm.

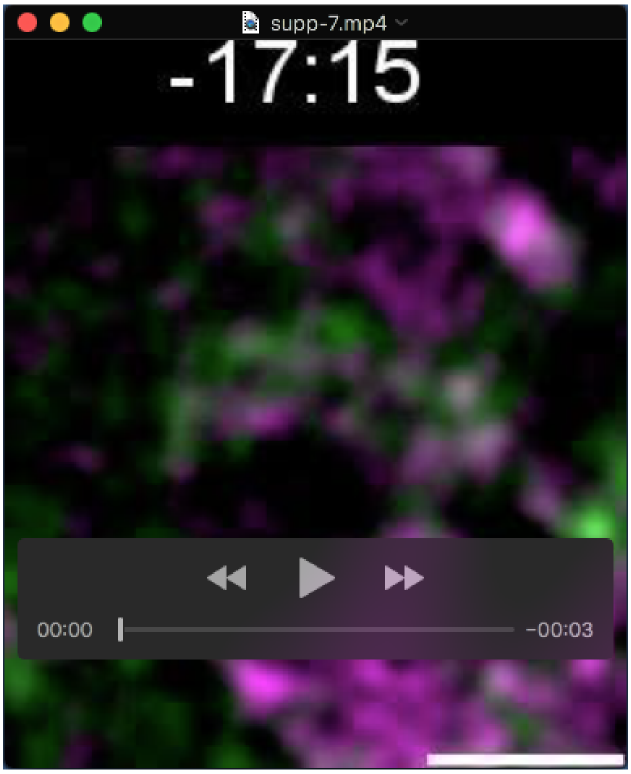

Movie 7 – *Embryonic apkc^{as4} mutant NB polarizes Baz and Mira normally before addition of 1NA-PP1 and polarizes Baz but not Mira after 1NA-PP1 addition*. Example of a aPKCas4 embryonic NB expressing Baz::GFP (green) and Mira::mCherry (magenta). Z stack collected every 2min30sec, movie displayed at 3 frames per sec. Time: mm:ss. 00:00 marks the addition of 20 μM 1NA-PP1. Scale bar: 5 μm.

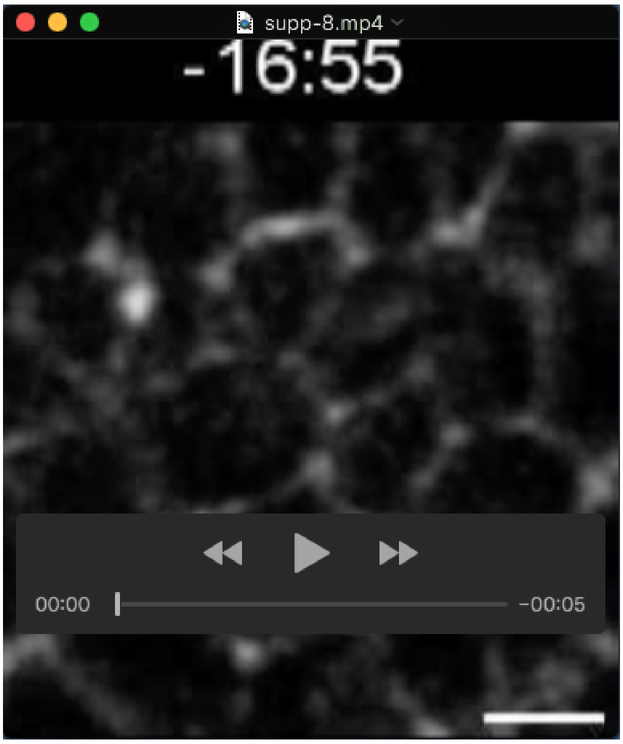

Movie 8 – *Embryonic apkc*^{as4} *mutant epithelial cells apically contract following 1NA-PP1 addition*. Example of adherens junctions of aPKCas4 embryonic epithelial cells expressing Baz::GFP. Z stack collected every 2min30sec, movie displayed at 3 frames per sec. Time: mm:ss. 00:00 marks the addition of 20 µM 1NA-PP1. Scale bar: 5 µm.

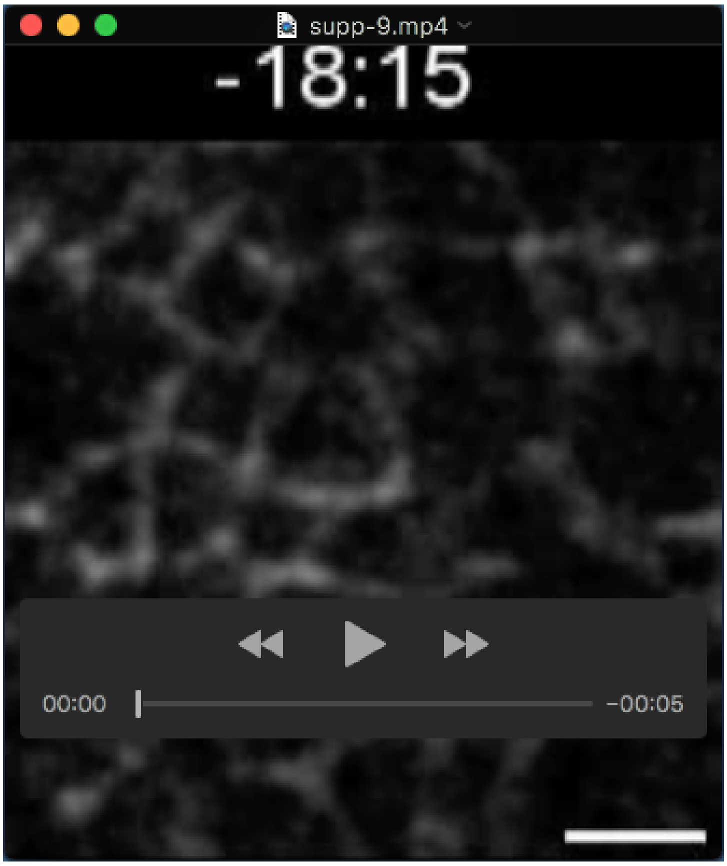

Movie 9 – *Control embryonic epithelial cells do not apically contract following 1NA-PP1 addition*. Example of adherens junctions of control embryonic epithelial cells expressing Baz::GFP. Z stack collected every 2min30sec, movie displayed at 3 frames per sec. Time: mm:ss. 00:00 marks the addition of 20 μM 1NA-PP1. Scale bar: 5 μm.

Development • Supplementary information

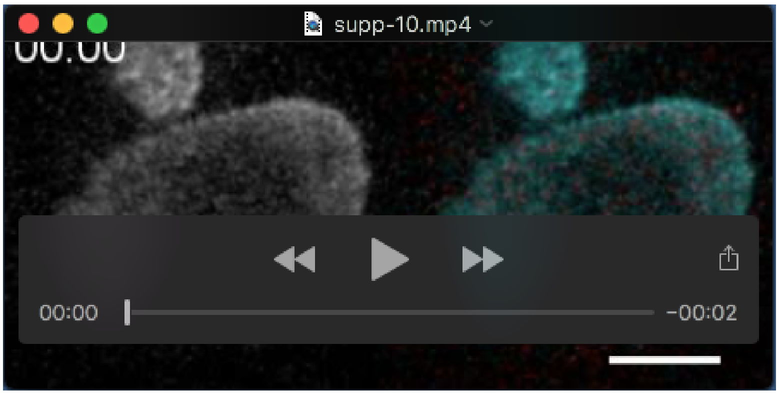

Movie 10 – *Acute inhibition of aPKC leads to loss of any asymmetric localization of Mira when* $apkc^{as4}$ *mutant larval NBs cycle into colcemid induced mitotic arrest*. in Example of an isolated $apkc^{as4}$ NB expressing Baz::GFP and Mira::mCherry. Left Panel shows Miranda (Grey). Right Panel shows Miranda (Cyan) and Baz (Red). NB enters mitosis in the presence of colcemid and 10µM 1NA-PP1. Z stacks collected every 2min, movie displayed at 5 frames per sec Time: hh:mm. Scale bar: 10µm.

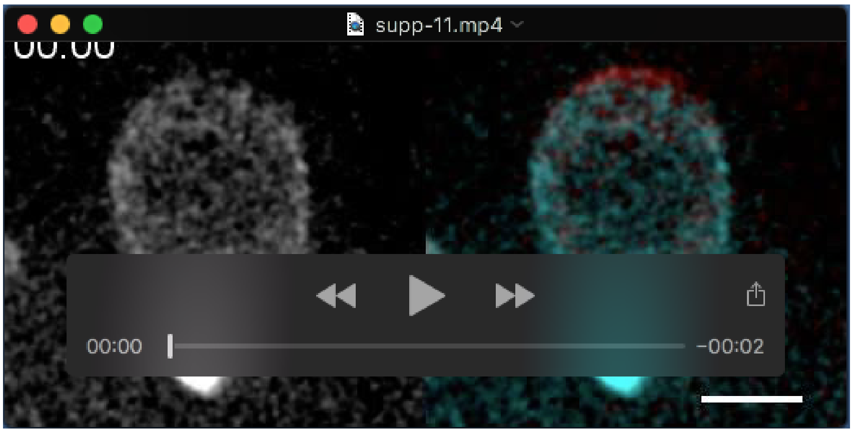

Movie 11 – *Control larval NBs in primary cell culture polarize Mira normally when cycling into colcemid induced arrest in the presence of 1NA-PP1*. Example of an isolated control NB expressing Baz::GFP and Mira::mCherry. Left Panel shows Miranda (Grey). Right Panel shows Miranda (Cyan) and Baz (Red). NB enters mitosis in the presence of colcemid and 10µM 1NA-PP1. Z stacks collected every 2min, movie displayed at 5 frames per sec. Time: hh:mm. Scale bar: 10µm.

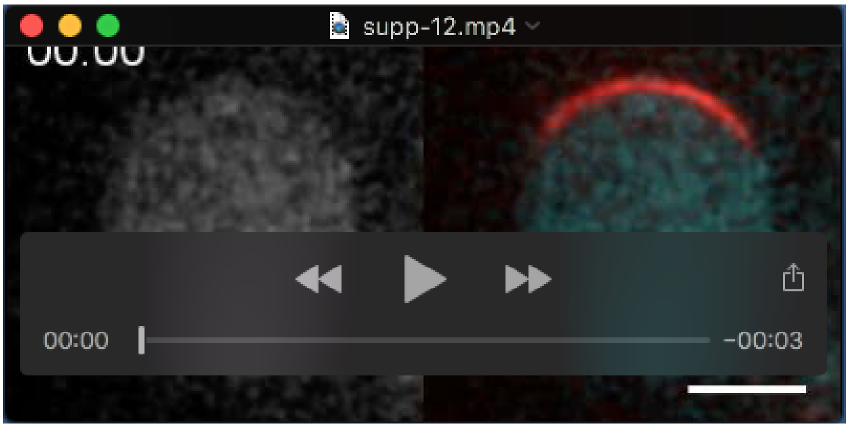

Movie 12 – *Basal Mira localization is unaffected by 1NA-PP1 addition in colcemid arrested control larval NBs upon addition of 1NA-PP1*. Example of an isolated control NB expressing Baz::GFP and Mira::mCherry. Left Panel shows Miranda (Grey). Right Panel shows Miranda (Cyan) and Baz (Red). NB arrested in mitosis with Colcemid prior to the addition of 10µM 1NA-PP1. Z stacks collected every 2min, movie displayed at 5 frames per sec. Time: hh:mm. Scale bar: 10µm.

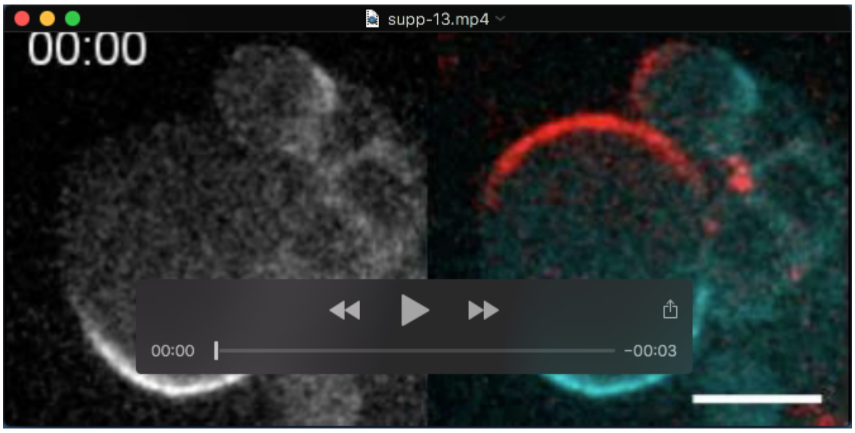

Movie 13 – *Mira continues to localize with a basal bias upon acute aPKC inhibition in apkc^{as4} mutant larval NBs in primary cell culture.* Example of an isolated *apkc^{as4}* NB expressing Baz::GFP and Mira::mCherry. Left Panel shows Miranda (Grey). Right Panel shows Miranda (Cyan) and Baz (Red). NB arrested in mitosis with Colcemid prior to the addition of 10µM 1NA-PP1. Z stacks collected every 2min, movie displayed at 5 frames per sec. Time: hh:mm. Scale bar: 10µm.

Development • Supplementary information

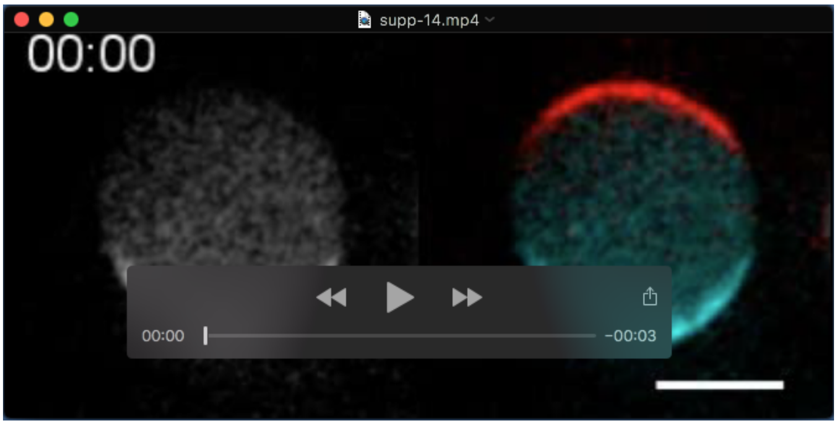

Movie 14 – *The basal bias of Mira localization upon acute aPKC inhibition in colcemid arrested larval NBs in primary cell culture is Lat-A sensitive.* Example of an isolated *apkc*^{as4} NB expressing Baz::GFP and Mira::mCherry. Left Panel shows Miranda (Grey). Right Panel shows Miranda (Cyan) and Baz (Red). NB arrested in mitosis with Colcemid prior to the addition of 10µM 1NA-PP1. Latrunculin-A was added after 45minutes. Z stacks collected every 2min, movie displayed at 5 frames per sec. Time: hh:mm. Scale bar: 10µm.

Development • Supplementary information
